# Migraine equivalents and related symptoms, psychological profile and headache features: which relationship?

**DOI:** 10.1186/s10194-015-0536-2

**Published:** 2015-06-09

**Authors:** Samuela Tarantino, Cristiana De Ranieri, Cecilia Dionisi, Valentina Gagliardi, Alessandro Capuano, Federico Vigevano, Simonetta Gentile, Massimiliano Valeriani

**Affiliations:** Headache Center, Division of Neurology, Ospedale Pediatrico Bambino Gesú, IRCCS, Piazza Sant’Onofrio 4, 00165 Rome, Italy; Unit of Clinical Psychology, Ospedale Pediatrico Bambino Gesù, IRCCS, Piazza Sant’Onofrio 4, Rome, Italy; Center for Sensory-Motor Interaction, Aalborg University, Aalborg, Denmark

**Keywords:** Children, Migraine equivalents, Psychological factors, Anxiety, Somatization, Hypochondria

## Abstract

**Background:**

Migraine equivalents are common clinical conditions in children suffering from headache. Very few studies dealt with the psychological profile of children/adolescents with migraine equivalents. Our main aim was to compare the psychological profile between migraine children with and without migraine equivalents. Moreover, as secondary aim, exclusively in children with migraine equivalents, we investigated the possible relationship between migraine attack frequency and intensity and psychological factors.

**Methods:**

We enrolled 136 young migraineurs. They were divided in two groups (patients with and without migraine equivalents). The psychological profile was assessed by means of SAFA Anxiety and Somatization questionnaires.

**Results:**

Migraine equivalents were present in 101 patients (74.3 %). Anxiety (*p* = 0.024) and somatization (*p* = 0.001) levels, but not hypochondria (*p* = 0.26), were higher in patients with migraine equivalents. In children with migraine equivalents, a low frequency of attacks was related to separation anxiety (*p* = 0.034).

**Conclusions:**

Migraine equivalents patients tend to feel more fearful and to experience more shyness. This, together with the tendency to somatization, may lead them to become vigilant in attachment relationships with their caregivers.

## Background

The clinical history of migrainous children is often characterized by symptoms that may precede or coexist with headache [1–6]. Known as “migraine equivalents” [3, 4], “childhood periodic syndromes” [2, 5, 6] and more recently “episodic syndromes” [1], symptoms described under this category are various and include a large range of clinical conditions. The International Classification of Headache Disorders, 3^rd^ edition (ICHD-III) describes only four migraine equivalents [1]. These are “Cyclical vomiting syndrome” (1.6.1.1), “Abdominal migraine” (1.6.1.2), “Benign paroxysmal vertigo (1.6.2) and “Benign paroxysmal torticollis” (1.6.3) [1]. There is still no agreement on the prevalence of migraine equivalents and related symptoms (MERS) and data depend on the population sample and/or included symptoms [3, 4]. Although not universally accepted, other clinical entities (e.g. motion sickness and limb pain) are very common among headache children [4–6]. In a recent study, we found a high prevalence (70.3 %) of MERS in children/adolescents suffering from primary headache with abdominal migraine (48.9 %), limb pain (43.9 %), and motion sickness (40.5 %) as the most common [4]. Moreover, the relationship between the frequency of headache attacks and the presence of MERS suggested that these symptoms are not only precursors of headache, but they can be considered as part of the migrainous syndrome in children [4].

Although the triggering factors of MERS are not well established, there is evidence that precipitating and relieving factors, including physical stress or psychological triggers, are often common to both headache attacks and MERS [2, 7, 8]. Comorbidity between childhood migraine and psychiatric disorders has been extensively studied [9–12]. In particular, anxiety symptoms are prevalent in children suffering from a high frequency migraine attacks [9, 11, 12]. On the contrary, little is known about the role of psychological factors in children with MERS. As headache attacks, also MERS are associated with internalizing disorders (such as anxiety and somatization) [13–17]. To the best of our knowledge, no study has compared the psychological profile between headache children with and without MERS.

In the present study we aimed to investigate the anxiety and somatization levels in migraine children suffering from MERS, as compared to those without MERS. Moreover, as secondary aim exclusively in the group of patients with MERS, the effect of anxiety and somatization on frequency and severity of headache attacks was investigated.

We hypothesized that: 1) patients with MERS have higher amount of psychological symptoms compared to children without MERS; 2) in children with MERS, anxiety levels and somatization are associated to headache severity (frequency/intensity of the attacks).

## Methods

### Patients and procedure

A total of 136 consecutive children/adolescents suffering from migraine without aura (MoA, ICHD-III) were included (67 males and 69 females; age range 8–17 years; mean age 11.5 ± 2.3 years). They were consecutively chosen from patients referred for consultation to our Headache Centre. Patients suffering from any other neurological or internal disease were excluded from our study.

At the time of the psychological evaluation, none of the patients were receiving drugs for migraine prophylaxis and none of them had been treated with other drugs acting on the central nervous system. Some children/adolescents had previously taken symptomatic drugs for pain relief.

At the initial visit, all patients were given a headache diary where they had to sign the main features of their headache for the next two months. Data on the clinical characteristics of migraine, including frequency and intensity of the attacks, were issued from the diary that was brought back at the second consultation (two months after the initial visit). Patients were divided in 2 groups according to headache attack frequency: 1) high frequency (HF) patients, having from weekly to daily episodes, and 2) low frequency (LF) patients, showing ≤ 3 episodes per month.

The cut point was chosen for three reasons: (1) patients with chronic and intermediate frequencies were too few to undergo reliable statistic comparison; 2) a mere distinction between chronic and episodic patients would have led to include individuals with high, but not chronic, headache episode frequency in the same group of patients with very low attack frequency; 3) the chosen cut point had the rationale to distinguish patients who need prophylactic treatment from those who do not.

According to headache attack intensity, patients were classified in 3 groups: (1) mild pain (MP), allowing the patient to continue his/her daily activities; (2) moderate pain (MoP) leading to interruption of patient activities; and (3) severe pain (SP), forcing the child to go to bed.

The MERS investigation was carried out by an interview during the initial assessment of the child. The interview was designed to provide sufficient information concerning the characteristics of the symptoms in order to allow them to be classified as MERS or otherwise. Possible organic causes of the symptoms, e.g., other neurological diseases for benign paroxysmal vertigo or gastroenterological abnormalities for abdominal migraine and cyclical vomiting, were investigated and their occurrence led to patient exclusion. Diagnosis was based on the presence of the typical clinical features of the MERS included in the ICHD-III (cyclical vomiting, abdominal migraine, benign paroxysmal torticollis and benign paroxysmal vertigo). Although not classified among the “episodic syndromes” in the International Classification, we also included limb pain and motion sickness, very common among headache children [4, 6]. The diagnosis of limb pain was based on the following criteria: 1) pain is usually non-articular, located in the lower extremities, and is usually bilateral; 2) pain appears late in the day or is nocturnal, often awaking the child; 3) parents often report pain on days of increased physical activity; 4) duration ranges from minutes to hours, and the intensity can be mild or very severe; 5) there are no objective signs of inflammation on physical examination; 6) limb pain are episodic, with pain free intervals from days to months [18]. The diagnosis of motion sickness was made in children experiencing discomfort when perceived motion disturbs the organs of balance; they could show nausea, vomiting, pallor, cold sweats, hypersalivation, hyperventilation and headaches [19]. Both patients who had complained MERS only in the past and those who continued to suffer from one or more MERS were included.

Psychological evaluation was performed in a single session by the same examiner (S.T.) with a specific training on the psychological assessment of children and adolescents. In order to exclude a possible direct effect of pain on psychological assessment, we ensured us that no patient had a headache attack within 24 h before the psychological study. All the patients were able to understand and to complete the tasks. None of them had ever had a previous psychological screening. The study was approved by the ethical committee of Bambino Gesù Children’s Hospital and all participants and their parents gave signed, informed consent to participate to the study.

### Psychological tool

Psychological tool employed in our study was the Italian SAFA battery of tests (Psychiatric scales for self-administration for youths and adolescents) [20, 21]. It allows examiners to explore a wide series of symptoms and psychological conditions. The entire battery includes a total of six scales (each with subscales) that can also be used separately. It evaluates anxiety-related areas (SAFA-A), depression-related areas (SAFA-D), obsessive–compulsive symptoms (SAFA-O), somatic concerns (SAFA-S), psychogenic eating disorders (SAFA-P) and phobias (SAFA-F). The administration lasts between 30 and 60 min. The SAFA battery is organized to fit the mode of understanding and evaluation of a large age group: each questionnaire is composed of a version for children aged from 8 to 10 years (identified with the letter “e”) and a version for subjects ranging from 11 to 18 years (identified with the letters “ms”). Only the scale for anxiety presents three distinct versions: 8–10 years (“e”), 11–13 years (“m”) and 14–18 years (“s”). There are three possible responses to each item: ‘true, false and partly true’; the sum of points achieved in each scale and subscale can be converted into *T* scores, sten points and percentiles. On the basis of the obtained scores, it is possible to build a general profile and/or individual profiles within the single scales. The scales showed good internal consistency (Cronbach alpha > 0.80) and test-retest stability. The psychometric properties have been established for each scale [20].

According to the aim of our study, we administered the scale for assessment of anxiety (SAFA-A) and somatization (SAFA-S). SAFA-A includes several subscales (“Generalized anxiety”, “Social anxiety”, “Separation anxiety”, “School anxiety”) and produces a “Total anxiety” score. SAFA-S considers somatic symptoms and hypochondria.

### Statistical analysis

Statistical evaluation was performed using Statistical Package for the Social Sciences (SPSS 18.0) software. According to the aims of our study, patients were divided in two groups (patients with and without MERS). Moreover, only the patients with MERS were grouped on the base of attack frequency (LF and HF groups) and headache pain severity (MP, MoP, and SP groups). Initially, we analyzed the frequencies of each category of variables (frequency, intensity). We used descriptive statistics expressed as means, SD and percentages to describe the basic features of our sample.

Analysis of variance (ANOVA) and *t*-test were used to estimate differences between group means. Data were analyzed in two stages: 1) migraine children with and without MERS were compared, and 2) in migraineurs with MERS, a series of one-way ANOVAs were carried out to further explore differences of anxiety and somatization, as function of the different levels of headache attack frequency and intensity. To assess whether there was a relationship between anxiety and somatization in our patients with MERS, we performed a series of correlation analyses between all SAFA-A and SAFA-S subscales. Pearson correlation coefficients with Bonferroni’s corrections for multiple comparisons were calculated.

The level of statistical significance was set at P < 0.05.

## Results

### Headache characteristics and migraine equivalents

Clinical characteristics of our patients are summarized in Table 1.

**Table 1:** Headache characteristics of our sample

	*N* = 136
Pain intensity	
Mild	32 (23.5 %)
Moderate	35 (25.8 %)
Severe	69 (50.7 %)
Frequency	
Low frequency	75 (55.1 %)
High frequency	61 (44.9 %)
Associated symptoms	
Nausea	49 (36 %)
Vomiting	32 (23.5 %)
Phonophobia	88 (64.7 %)
Photophobia	83 (61 %)

Headache pain rating was severe, moderate, and mild in 50.7 %, 23.5 %, and 25.8 % of patients, respectively. Most patients (55.1 %) had low frequency headache attacks, whereas 44.9 % complained of high frequency episodes.

Migraine equivalents were reported by 74.3 % of patients. Motion sickness (42.6 %), limb pain (37.5 %), and abdominal migraine (40.4 %) were the most common MERS (Table 2). Many patients (44.8 %) complained of more than one MERS. Among them, 45.5 % suffered from two MERS, whereas 14.8 % had three or more MERS. Clinical characteristics of children/adolescents with and without MERS are described in Table 3.

**Table 2:** Migraine equivalents distribution among our patients

	Number	Percent
Motion sickness	58	42.6 %
Limb pain	51	37.5 %
Abdominal migraine	55	40.4 %
Cyclic vomiting	5	3.7 %
Benign paroxismal vertigo	11	8.1 %
Benign paroxismal torticollis	0	0 %

**Table 3:** Headache characteristics in children with and without migraine equivalents

	Patients with migraine equivalents (n.101)
Pain intensity	
Mild	24 (23.8 %)
Moderate	24 (23.8 %)
Severe	53 (52.4 %)
Frequency	
Low frequency	59 (58.4 %)
High frequency	42 (41.6 %)
Associated symptoms	
Nausea	44 (43.6 %)
Vomiting	26 (25.7 %)
Phonophobia	65 (64.4 %)
Photophobia	67 (66.3 %)
	Patients without migraine equivalents (n. 35)
Pain intensity	
Mild	8 (22.9 %)
Moderate	11 (31.4 %)
Severe	16 (45.7 %)
Frequency	
Low frequency	16 (45.7 %)
High frequency	19 (54.3 %)
Associated symptoms	
Nausea	14 (40.0 %)
Vomiting	9 (25.7 %)
Phonophobia 64	23 (65.1 %)
Photophobia	19 (54.3 %)

### Anxiety and somatization levels in patients with and without migraine equivalents

In both SAFA-A and SAFA-S total scores, patients with MERS showed worse values than those without MERS (SAFA-A Tot: F_(1, 134)_ = 12.16, *p* = 0.001; SAFA-S Tot: F_(1, 134)_ = 5.18, *p* = 0.024). While all SAFA-A subscales were higher in patients with MERS (p < 0.05), only the “Somatic symptoms” among SAFA-S subscales was higher in children with MERS (F_(1, 134)_ = 4.96; *p* = 0.028). No significant difference between patients with and without MERS was found in in “Hypochondria” subscale of SAFA-S (F_(1, 134)_ = 1.23; *p* = 0.26) (Table 4).

**Table 4:** Anxiety and somatization symptoms in patients with and without migraine equivalents

	With migraine equivalents	Without migraine equivalents	
SAFA scales	Mean ± SD	Mean ± SD	P
SAFA-A Generalized anxiety	9.7 ± 5.1	6.7 ± 4.4	0.001*
SAFA-A Social anxiety	7.1 ± 4.0	4.9 ± 3.3	0.004*
SAFA-A Separation anxiety	7.6 ± 4.9	5.3 ± 4.5	0.020*
SAFA-A Scholastic anxiety	9.4 ± 5.4	6.8 ± 4.3	0.009*
SAFA-A Total anxiety	33.5 ± 14.9	23.7 ± 12.7	0.001*
SAFA-S Somatic symptoms	13.4 ± 5.6	11.1 ± 3.6	0.028*
SAFA-S Hypochondria	1.4 ± 1.4	1.1 ± 1.1	0.260
SAFA-S Total somatization	14.8 ± 6.0	12.4 ± 4.3	0.024*

*P ≤ 0.05

### Headache features, anxiety and somatization in patients with migraine equivalents

In patients with MERS, attack frequency, but not intensity, showed a significant effect on anxiety symptoms. In particular, the LF group had higher “Separation anxiety” scores than HF patients (F_(1, 99)=_4.63; *p* = 0.034). SAFA-S values, were not different between the two frequency groups (SAFA-S Tot, F_(1, 99)_ = 1.21; *p* = 0.26) (Table 5).

**Table 5:** SAFA-A and SAFA-S raw scores (mean ± standard deviation) and ANOVA among pain intensity-based groups (migraineurs with MERS)

SAFA scales	MP	MoP	SP	*F* value	P
SAFA-A Ge	10.0 ± 4.6	11.2 ± 5.9	8.8 ± 4.9	1.92	0.15
SAFA-A So	6.7 ± 4.2	7.6 ± 4.2	7.1 ± 3.8	0.35	0.71
SAFA-A Se	8.3 ± 4.7	6.8 ± 4.7	7.6 ± 5.0	0.58	0.55
SAFA-A Sc	8.9 ± 5.7	10.1 ± 5.6	9.3 ± 5.2	0.32	0.72
SAFA-A Tot	33.6 ± 16.0	35.5 ± 14.7	32.6 ± 14.6	0.32	0.72
SAFA-S So	13.0 ± 4.0	15.1 ± 5.3	12.8. ± 6.3	1.46	0.24
SAFA-S Hy	1.7 ± 2.2	1.7 ± 1.1	1.2 ± 1.1	1.53	0.22
SAFA-S Tot	14.7 ± 4.4	16.7 ± 6.0	14.0 ± 6.8	1.70	0.19

MP mild pain; MoP moderate pain; SP severe pain intensity; SAFA, Psychiatric scales for self-administration for youths and adolescents; SAFA-A Ge, “Generalized anxiety” subscale; SAFA-A So, “Social anxiety” subscale; SAFA-A Se, “Separation anxiety” subscale; SAFA-A Sc, “School anxiety” subscale; SAFA-A Tot, “Total anxiety” scale; SAFA-S So, “Somatic symptoms” subscale; SAFA-S Hy, “Hypochondria”; SAFA-S Tot, “Total Somatization”

*P ≤ 0.05

No significant effect of pain intensity on the main SAFA-A (SAFA-A Tot: F_(2, 98)_ = 0.32, *p* = 0.72) and SAFA-S (SAFA-S Tot, F_(2, 98)_ = 1.70; *p* = 0.19) scales was found (Table 6).

**Table 6:** SAFA-A and SAFA-S raw scores (mean ± standard deviation) and ANOVAs among frequency-based groups (migraineurs with MERS)

SAFA scales	LF	HF	*F* value	P
SAFA-A Ge	9.6 ± 4.9	9.8 ± 5.5	0.034	0.84
SAFA-A So	7.2 ± 3.7	7.0 ± 4.4	0.25	0.81
SAFA-A Se	8.4 ± 5.1	6.3 ± 4.3	4.63	0.034*
SAFA-A Sc	9.8 ± 5.3	8.9 ± 5.5	0.65	0.41
SAFA-A Tot	34.8 ± 15.0	31.8 ± 14.7	1.01	0.32
SAFA-S So	13.8 ± 6.1	12.8 ± 5.0	0.74	0.38
SAFA-S Hy	1.6 ± 1.7	1.2 ± 0.8	1.84	0.18
SAFA-S Tot	15.4 ± 6.6	14.0 ± 5.4	1.21	0.26

LF, low frequency; HF, high frequency; SAFA, Psychiatric scales for self-administration for youths and adolescents; SAFA-A Ge, “Generalized anxiety” subscale; SAFA-A So, “Social anxiety” subscale; SAFA-A Se, “Separation anxiety” subscale; SAFA-A Sc, “School anxiety” subscale; SAFA-A Tot, “Total anxiety” scale; SAFA-S So, “Somatic symptoms” subscale; SAFA-S Hy, “Hypochondria”; SAFA-S Tot, “Total Somatization”

*P ≤ 0.05

In patients with MERS, the anxiety symptoms showed a relationship with the somatization level. In particular, a negative and significant correlation emerged between “Separation anxiety” subscale (SAFA-A Se) and “Somatic symptoms” subscale (SAFA-S Som) (r = −0.18; *p* = 0.033). “Generalized”, “Social” and “Scholastic” anxiety scores did not correlate with any SAFA-S subscale.

## Discussion

The main results of the present study is that young migraineurs with MERS have higher symptoms of anxiety and somatic complaints than those without MERS. Moreover, we found that in patients with MERS a low attack frequency is related to “Separation anxiety” and there is a negative correlation between “Separation anxiety” and “Somatic complaints”.

### Migraine, migraine equivalents and anxiety

Among our migraine patients, those with MERS had higher anxiety levels, as compared to patients without MERS. Anxiety symptoms were higher in all of SAFA-A subscales indicating that patients with MERS experience more anxious feelings in several fields such as school, social relationships, and separation from parents.

Very few studies analyzed the psychological profile in children with MERS and they focused only on cyclical vomiting syndrome and benign paroxysmal vertigo [13–17]. Previous findings indicated that there is a high prevalence of internalizing disorders, especially anxiety symptoms in children with cyclic vomiting syndrome [13, 14, 17]. High levels of anxiety and somatization were recorded in children benign paroxysmal vertigo [15, 16]. The relationship between headache, somatic complaints and the psychological profile has been explored rarely [12, 22]. Moreover, these previous studies did not include MERS according to ICHD-III [1] and were not conducted on a selected population of migraine children/adolescents.

The relationship between migraine, MERS and psychiatric disorders seems very complex. Results of longitudinal studies suggest that the association between headache and psychological distress can be bi-directional [10, 23]. In pediatric age, migraine could be worsened by psychiatric condition and, on the other hand, migraine itself could be considered a source of stress that, in turn, may cause emotional symptoms [9]. The observed association among migraine, MERS, and anxiety symptoms found in our study raises further questions of whether the relationship may be due to a shared diathesis with common underlying risk factors. The presence of anxiety symptoms in MERS patients could be attributable to a common mechanism underlying the three conditions (migraine, MERS, and anxiety). Several neurotransmitters (such as serotonin), which are classically considered to play a primary role in migraine pathophysiology [24, 25], are also know to contribute to the pathogenesis of some functional disorders, including recurrent abdominal pain [26], motion sickness [25], and anxiety [27]. According to these data, our findings expand and compliment the hypothesis that MERS are not only precursors of headache, but they are part of the migrainous syndrome in children [4].

### Migraine, migraine equivalents and somatization

In the present study, patients with MERS reported more frequently somatic complaints, but not hypochondria, as compared to those without MERS. Hypochondria—or, as it is now defined, “illness anxiety disorder” (DSM-V) [28]- is a type of anxiety characterized by excessive preoccupancy or worry about having a serious illness. Patients suffering from hypochondria may or may not have a medical condition, but they have heightened bodily sensations. Hypochondria represents a predictor for higher disability in adult headache [29]. In particular, it has been suggested that high “anxiety sensitivity”, through a misinterpretation of innocuous sensation, may cause a sympathetic arousal, which in turn, lead to the headache attack [30]. So far, no study has analyzed hypochondria levels in MERS children/adolescents. We can hypothesize that in our patients with MERS the higher level of somatic complaints is not related to concerns about physical health or an inaccurate perception of body signs, but to a tendency to express negative emotions, such as anxiety and stress, through somatic complaints. These results are consistent with the psychosomatic explanation for MERS proposed by some authors [6, 31]. In particular, in a previous study by Lanzi et al., an overlapping prevalence of periodic symptoms in migraineurs children and psychosomatic patients was found [6]. The author suggested that periodic syndromes may be predictive of the subsequent development of a psychosomatic pathology. However, more clinical investigation is needed to confirm this relationship.

### Relationship between separation anxiety, somatic symptoms and headache feature in patients with MERS

In our patients with MERS, “Separation anxiety” subscale had the highest scores in those with a low attack frequency. Moreover, separation anxiety symptoms showed a negative correlation with SAFA-S “Somatic symptoms” subscale. Separation anxiety disorder (SAD) is an anxiety disorder of middle childhood characterized by an excessive worry about separation from another person, typically a parent, who represents safety for the affected child [32]. Somatic symptoms such as headache, nausea and more often abdominal pain are common features of SAD [32]. It is important to differentiate SAD from other anxiety disorders. Children with other anxiety disorders also might fear separation from parents, but their fears are based on different concerns. There is a growing body of literature reporting a high pain intensity and disability in children with SAD symptoms [33, 34]. While the relationship between anxiety and headache has been repeatedly investigated in both adult [10, 23] and pediatric patients [10, 11], possible correlations between separation anxiety symptoms and migraine has been rarely considered and data are so far inconclusive [21, 22, 35]. To the best of our knowledge, no study dealt with separation anxiety in headache children with migraine equivalents. Our findings on MERS patients, confirmed what we have already shown in a previous study [21], suggesting that the higher is the separation anxiety, the lower is the headache attack frequency. The “Attachment theory” can provide a theoretical base for understanding our results. We can hypothesize that patients with MERS, being more anxious and less confident with themselves, tend to be more dependent on others. This, together with the tendency to report more somatic complaints, may lead them to become vigilant in attachment relationships, seeking support in response to stressful situations. We can suppose that in our patients a low headache attack frequency may reduce the proximity and the attention from the caregiver, thus it can lead the separation anxiety feelings to be increased.

### Limitations of the study

This study has a number of limitations that must be taken into account in interpreting the results.

1) Our findings are issued from children referred to our third-level headache center, thus they may not be representative of the general population. 2) Since the patients and their parents were asked to report the MERS symptoms once and retrospectively, there is a risk for biased estimates of the various complaints. Moreover, given the lack of inclusion criteria for limb pain and motion sickness in ICHD-III, the diagnosis of these disorders was mainly based on the exclusion of other disorders. 3) The SAFA-A and S tests, used to investigate anxiety and somatization, have a fundamental self-report nature. While these questionnaires have been shown to be valid instruments for screening children with psychiatric disorders, formal diagnosis of psychiatric disorders cannot be inferred. 4) Since all the children included in our study were migraineurs, one may wonder whether the psychological elements described in our patients were linked to migraine rather than MERS. However, migraine was shared by both groups of our patients, who only differed for the presence/absence of MERS. Therefore, we can exclude that the psychological difference between groups is due to migraine. In future studies, it will be interesting to investigate the psychological difference between children with only migrainous headache and those with only MERS.

## Conclusions

Our data indicate that symptoms of anxiety and somatization are associated with migraine equivalents in children/adolescents suffering from migraine. These results suggest that a systematic evaluation of children and adolescents with MERS should include a psychological screening, due to the potential impact that these comorbid psychological symptoms can have on their clinical outcomes.

## Abbreviations

MERS: Migraine equivalents and related symptoms
ICHD-III: Headache Classification Committee of the International Headache Society
HF: High frequency
LF: Low frequency
MP: Mild pain
MoP: Moderate
SP: Severe pain
SAFA: Psychiatric scales for self-administration for youths and adolescents
SAD: Separation anxiety disorder
